# Consistency of Intraoral-Scanner-Derived Occlusal Contact Distance Maps Between Centric Relation and Maximal Intercuspation

**DOI:** 10.3390/dj14040199

**Published:** 2026-04-01

**Authors:** Dubravka Knezović Zlatarić, Maja Žagar

**Affiliations:** Department of Removable Prosthodontics, School of Dental Medicine, University of Zagreb, Gundulićeva 5, 10000 Zagreb, Croatia; mpavic@sfzg.hr

**Keywords:** intraoral scanner, digital occlusion, occlusal contact distance, centric relation, maximal intercuspation, occlusal analysis, image segmentation

## Abstract

**Background/Objectives:** Intraoral scanners (IOSs) are increasingly used for digital occlusal evaluation; however, the interpretation of IOS-derived occlusal contact distance visualizations remains influenced by registration and alignment factors. This study aimed to compare occlusal contact distance distributions obtained with an intraoral scanner between maximal intercuspation (MIP) and centric relation (CR) under controlled scanning conditions. **Methods:** Fifteen participants underwent standardized digital bite registration in both mandibular positions. Only scans exhibiting interocclusal overlap ≤ 0.1 mm were included. Occlusal contact distance maps were exported, standardized, and quantified using an open-source image segmentation workflow to calculate the relative area of distance-based contact categories. Paired statistical analyses were performed to compare CR and MIP distributions. **Results:** No statistically significant differences were observed in the distribution of occlusal contact distance categories between centric relation and maximal intercuspation (*p* > 0.05). The proportion of very close contact distances remained low in both positions (approximately 6–9%), while moderately close and distant contact distances predominated across all scans, indicating comparable distance-based occlusal visualization patterns under controlled acquisition conditions. **Conclusions:** Under controlled scanning conditions, IOS-derived occlusal contact distance maps exhibited comparable distribution patterns between centric relation and maximal intercuspation. These findings support the use of intraoral scanning for comparative evaluation of occlusal contact distance distributions, while highlighting the importance of interpreting IOS-derived visualizations within their methodological and technical limitations.

## 1. Introduction

In modern dental practice, intraoral scanners (IOSs) are routinely used for diagnostics, treatment planning, and the fabrication of restorations due to having several advantages over conventional impression techniques, including improved patient comfort, reduced clinical time, digital data storage, real-time visualization, and streamlined communication with dental laboratories [1,2,3,4,5].

Despite these advantages, digital bite registration remains a technically sensitive step within IOS workflows, particularly when assessing occlusal relationships. Typically, the maxillary and mandibular arches are scanned separately and subsequently aligned using a buccal scan performed in maximal intercuspation. This indirect alignment process relies on algorithmic matching of scanned surfaces and may be influenced by factors such as mandibular repositioning, soft-tissue resilience, and scan alignment variability. As a result, discrepancies in the spatial relationship between virtual arches may occur and affect the visualization of occlusal contacts [6,7,8,9].

Importantly, occlusal contact visualization generated by IOS software represents distance-based approximations between opposing virtual surfaces rather than direct measurements of occlusal force or pressure. Thus, the color-coded occlusal maps generated during digital bite registration reflect the ranges of interocclusal proximity and should be interpreted as relative contact distance information rather than biomechanical contact intensity. While such visualizations are commonly used in clinical workflows, their interpretation is inherently limited by the absence of true interocclusal force measurement [10].

Previous studies have evaluated aspects of digital occlusal analysis using scanned casts or intraoral models and have reported varying levels of accuracy and reproducibility depending on the methodology employed [11,12,13,14]. However, quantitative data on the consistency and comparability of IOS-derived occlusal contact distance distributions across different clinically relevant mandibular positions—such as centric relation (CR) and maximal intercuspation (MIP)—remain limited. Because both centric relation and maximal intercuspation are commonly used as clinical reference positions, assessing the comparability of IOS-derived occlusal contact distance distributions across these positions is important for the interpretability of digital bite registrations. In addition, practical and accessible workflows for standardized, image-based quantification of these distance maps remain insufficiently explored.

The aim of this study was to evaluate the consistency of occlusal contact distance maps generated by a single intraoral scanner by comparing the distribution of contact proximity categories between centric relation (CR) and maximal intercuspation (MIP) under controlled scanning conditions. In addition, the feasibility of using open-source image segmentation software for quantitative analysis of IOS-derived occlusal contact distance maps was assessed.

Research Hypotheses

• There is no statistically significant difference in the distribution of occlusal contact distance categories between centric relation (CR) and maximal intercuspation (MIP), as derived from intraoral-scanner-generated occlusal contact distance maps and quantified using image segmentation.
• The proportion of very close occlusal contact distances remains comparable between CR and MIP under controlled scanning conditions.
• The overall distribution of occlusal contact distance categories (very close, moderately close and distant contacts), as defined by the intraoral scanner’s color-mapping algorithm, demonstrates consistent patterns between CR and MIP, supporting the consistency of IOS-derived occlusal contact distance visualization.

## 2. Materials and Methods

### 2.1. Study Design and Sample Selection

This within-subject comparative study initially recruited 16 adult participants. One participant was excluded due to excessive interocclusal overlap (>0.1 mm) identified during scan quality control on the IOS-derived occlusal contact distance map, resulting in a final sample of 15 participants included in the analysis. The inclusion and exclusion criteria are summarized in Table 1. Participants were recruited during routine clinical examination and provided written informed consent prior to participation. The study was approved by the Ethics Committee of the School of Dental Medicine, University of Zagreb, Croatia (protocol code: 03-01/25-05/13, approval date: 12 November 2025).

### 2.2. Occlusal Position Registration

Each participant underwent intraoral scanning in both maximal intercuspation (MIP) and centric relation (CR). MIP was recorded during natural maximum voluntary closure, with participants instructed to close into their habitual intercuspal position to ensure a standardized mandibular position during scan acquisition [15].

Centric relation (CR) was registered using a Lucia jig following neuromuscular deprogramming and bilateral manipulation. During CR acquisition, participants were instructed to close lightly without active muscular contraction, allowing guided positioning into a clinically defined mandibular reference position suitable for digital bite registration [16].

To minimize potential registration artifacts related to virtual arch alignment, only scans demonstrating ≤ 0.1 mm occlusal overlap on the IOS-derived occlusal contact distance map were included in the analysis. This study design did not include repeated registrations within the same occlusal position and therefore does not assess test–retest repeatability in a metrological sense.

### 2.3. Digital Acquisition Protocol

All scans were obtained using the Medit i700 wired intraoral scanner (Medit Corp., Seoul, Republic of Korea). The maxillary and mandibular arches were scanned separately following the manufacturer’s recommended scanning sequence, after which a dedicated bite scan was captured to algorithmically align the two datasets and establish the virtual interocclusal relationship.

Occlusal contact distance maps were generated using the Medit Occlusal Analyzer module (Medit i700; Medit, Seoul, Republic of Korea) integrated within the Medit software(Medit Link v3.4.7; Medit Scan for Clinics v1.13.7) environment. All datasets were processed in Medit Link version 3.2.1 together with the Occlusal Analyzer application version 1.0.2, which represented the most recent software versions available at the time of data acquisition.

### 2.4. Image Analysis Protocol

**Figure 1:**
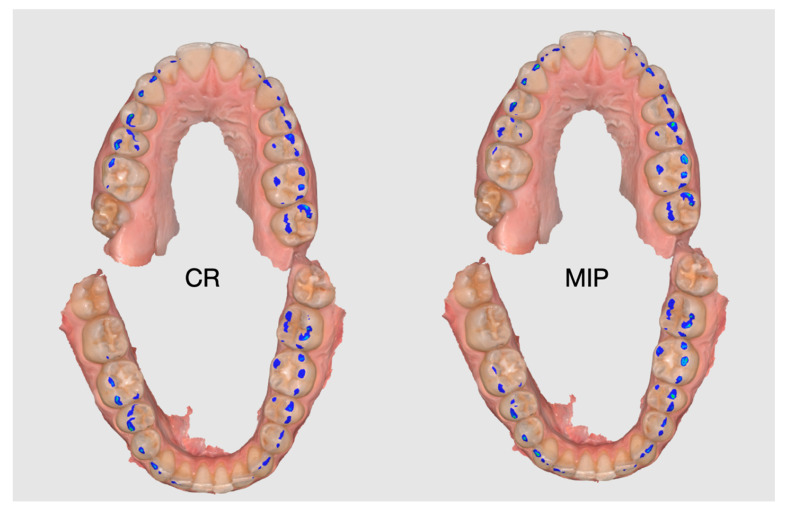
Representative examples of intraoral-scanner-derived occlusal contact distance maps obtained in centric relation (CR) and maximal intercuspation (MIP) from the same participant. Color-coded regions represent scanner-generated interocclusal distance categories and are shown in the “opened jaws” view.

All occlusal contact images were exported from the Medit Occlusal Analyzer using an identical “opened jaws” visualization, fixed screen magnification, and maximum display resolution, and were subsequently standardized prior to image analysis. Representative examples of exported occlusal contact distance maps in maximal intercuspation (MIP) and centric relation (CR) before segmentation are shown in Figure 1. Each image was captured as a full-screen screenshot at maximum display resolution and imported into GIMP 2.10 (GNU Image Manipulation Program). Images were converted to 300 dpi and cropped to a fixed dimension of 700 × 900 pixels to ensure a consistent field of view, including only the occlusal surfaces and corresponding color-coded contact distance areas.

Color-based segmentation was performed in GIMP using the Select-by-Color tool to isolate occlusal contact distance categories corresponding to the red, green, and blue ranges of the Medit color-mapping scale, representing very close, moderately close, and distant interocclusal contact distances, respectively. Color selection thresholds were applied consistently across all images to ensure the consistency of pixel selection. For each segmented color region, the total number of selected pixels was recorded, and the percentage of image area occupied by each distance category was calculated relative to the total pixel count (630,000 pixels).

A descriptive threshold of 15% was applied to the proportion of very close contact distances for comparative purposes only and did not represent a biomechanical or clinical force threshold.

To avoid interference from soft-tissue coloration, only occlusal surfaces were included in the analysis, and red-hued gingival regions were excluded from segmentation. All analyses were performed by a single calibrated operator following a standardized workflow to minimize observer-related variability.

For analytical purposes, the red, green, and blue distance ranges were subsequently referred to as very close, moderately close and distant occlusal contact distance categories, respectively.

### 2.5. Color Segmentation and Pixel Counting

All occlusal contact images were exported from the Medit Occlusal Analyzer in a standardized “opened jaws” view and processed using an identical workflow. Screenshots were imported into GIMP 2.10, converted to 300 dpi, and cropped to a fixed analysis frame of 700 × 900 pixels (630,000 pixels total), ensuring a uniform region of interest across all participants. The color selection parameters were established during preliminary testing to ensure consistent segmentation across all images.

Color-coded occlusal contact distance categories were segmented using the Select-by-Color tool in GIMP. Within the Medit color scale, red, green, and blue markings represent very close, moderately close, and distant interocclusal contact distances, respectively. Consistent threshold parameters were applied across all images to isolate each distance category while preserving pixel-level accuracy. The total number of selected pixels in each category was recorded and is expressed as a percentage of the standardized analysis frame.

Gingival areas were excluded prior to segmentation to prevent soft-tissue coloration from influencing measurements. This standardized image-processing workflow was designed to ensure consistent quantification of occlusal contact distance distributions across both CR and MIP scans.

### 2.6. Limitations Due to Pixel Overlap

Because color segmentation in GIMP is based on selecting pixels that fall within defined color ranges, partial overlap between color categories may occur when pixels contain mixed or transitional hues. As a result, some pixels may be included in more than one contact distance category, and the summed percentages of red, green, and blue areas may exceed 100%. This approach therefore does not produce mutually exclusive segmentation but provides a consistent estimate of relative contact distance distribution. Since the objective of the study was to compare distribution patterns between CR and MIP under identical image-processing conditions, this limitation does not affect the comparative consistency of the analysis.

### 2.7. Statistical Analysis

Descriptive statistics (means, standard deviations, and 95% confidence intervals) were calculated for occlusal contact distance category percentages in both CR and MIP. Normality assumptions were assessed prior to parametric testing. Differences in the proportion of very close occlusal contact distances (corresponding to the red range of the Medit color scale) between CR and MIP were evaluated using a paired-samples *t*-test, reflecting the within-subject design of the study.

A one-sample *t*-test was applied to descriptively assess whether the proportion of very close occlusal distance area differed from the predefined reference value of 15%, which was used for comparative purposes only and did not represent a clinical or biomechanical threshold. Effect sizes were reported using Cohen’s d. Statistical significance was set at *p* < 0.05. All analyses were performed using IBM SPSS 20.0 Statistics for Windows (IBM Corp., Armonk, NY, USA).

## 3. Results

**Figure 2:**
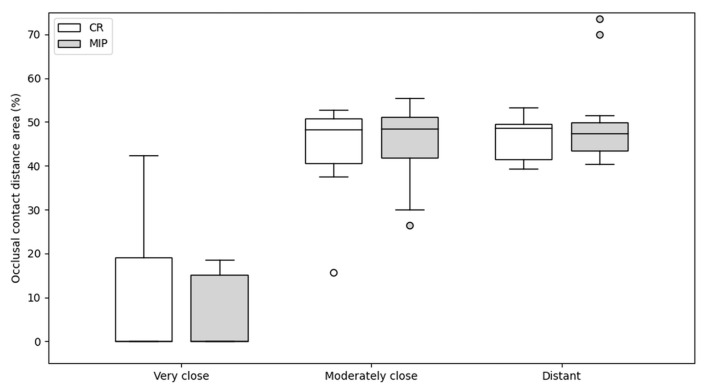
Distribution of occlusal contact distance area percentages for very close, moderately close, and distant contact distance categories in centric relation (CR) and maximal intercuspation (MIP). Categories correspond to the red, green, and blue ranges of the intraoral scanner color scale.

Fifteen paired intraoral scans were analyzed in both centric relation (CR) and maximal intercuspation (MIP). Although the segmentation approach can yield non-mutually exclusive values, minimal overlap was observed in the present dataset, and summed category percentages were close to 100%. Occlusal contact distance categories corresponding to the red, green, and blue ranges of the Medit color scale—representing very close, moderately, and distant occlusal contact distance categories, respectively—were quantified within a standardized pixel-based analysis frame. For comparative visualization purposes, values are reported as relative percentages (Figure 2).

The mean proportion of very close occlusal contact distances was 8.58% in CR and 5.92% in MIP. A paired-samples *t*-test showed no statistically significant difference between CR and MIP for this distance category (t = 0.84, *p* = 0.42).

Moderately close contact distances represented 44.78% of the occlusal surface in CR and 44.75% in MIP, with no statistically significant difference between positions (t = 0.01, *p* = 0.99).

Distant contact distances accounted for 46.63% of the occlusal surface in CR and 49.33% in MIP, also without a statistically significant difference (t = −1.02, *p* = 0.33).

Overall, the distribution of occlusal contact distance categories remained comparable between CR and MIP across all distance ranges (Figure 2; Table 2).

## 4. Discussion

The present study evaluated the distribution of intraoral-scanner-derived occlusal contact distance categories obtained in centric relation (CR) and maximal intercuspation (MIP) under standardized scanning conditions. No statistically significant differences were observed between CR and MIP for any of the analyzed contact distance categories, indicating comparable distribution patterns across the two mandibular positions.

These findings suggest that, within the methodological framework of this study, IOS-based occlusal contact distance maps provide similar distance-based visual representations when different mandibular reference positions are used. However, it should be emphasized that such visualizations are based on algorithm-derived interocclusal distance approximations rather than direct measurements of occlusal force and should be interpreted accordingly.

The first hypothesis addressed whether the distribution of very close occlusal contact distances differed between CR and MIP. This hypothesis was not rejected, as the proportion of very close contacts remained low in both mandibular positions (8.6% in CR and 5.9% in MIP), with no statistically significant difference between conditions.

Comparable distribution tendencies between different mandibular positioning protocols have also been reported in previous studies evaluating digital interarch alignment and virtual occlusal records, particularly when controlled mandibular guidance was applied during bite registration [17,18,19].

The second hypothesis examined whether the proportion of very close occlusal contact distances remained comparable between centric relation (CR) and maximal intercuspation (MIP). The observed proportions of very close contact distances were consistently low in both mandibular positions.

These findings suggest that, under the applied scan quality control criteria—specifically the exclusion of scans exhibiting excessive virtual overlap (>0.1 mm)—very close contact distances were not disproportionately represented in either CR or MIP. Similar distribution patterns have been reported in previous studies evaluating digital occlusal analysis workflows, in which the majority of detected contacts correspond to moderate or greater interocclusal separation, while very close contact distances account for a smaller proportion of the overall occlusal surface [20].

The third hypothesis examined whether the overall distribution of occlusal contact distance categories would demonstrate comparable patterns across centric relation (CR) and maximal intercuspation (MIP). In the present study, the occlusal surface in both mandibular positions was predominantly characterized by moderately close and distant interocclusal contact distances, while very close contact distances accounted for a smaller proportion of the total distribution.

Comparable distribution tendencies have been reported in previous studies evaluating digital occlusal analysis systems, which describe a predominance of non-contact or moderately close proximity zones in virtual occlusal maps, despite differences in system-specific algorithms and visualization parameters [21,22]. Systematic reviews further emphasize that IOS-derived occlusal contact visualizations should be interpreted as relative spatial representations rather than absolute measures of contact magnitude or biomechanical force [6].

Previous studies comparing intraoral-scanner-based occlusal analysis with articulating paper have reported that IOS-derived visualizations typically identify fewer apparent contact areas, reflecting differences in detection principles and visualization thresholds rather than direct equivalence with force-based occlusal assessment methods [23,24].

Beyond addressing the specific study hypotheses, the present findings contribute to the broader methodological discussion on digital occlusal analysis using intraoral scanners. Although IOS technology continues to evolve, the interpretation of occlusal contact distance maps remains influenced by multiple technical and procedural factors related to data acquisition, visualization algorithms, and study design. These factors should therefore be carefully considered when evaluating IOS-derived occlusal contact visualizations in both research and clinical contexts.

A central issue in the literature is the variability introduced by differing occlusal registration protocols, segmentation workflows, and scanner algorithms. Several studies have shown that IOS-derived occlusal contact representations are not strictly comparable across scanner platforms due to differences in image processing, contact-detection parameters, and the computational alignment of maxillary and mandibular datasets [6]. These methodological inconsistencies may contribute to the heterogeneity reported in comparative studies, particularly where direct numerical agreement between digital and physical methods is sought.

Within this context, the controlled scanning conditions applied in the present study—most notably the exclusion of scans exhibiting interocclusal overlap greater than 0.1 mm—should be regarded as a methodological strategy to limit alignment-related variability rather than as a validation of occlusal accuracy.

It should be noted that only a single centric relation (CR) and maximal intercuspation (MIP) registration was acquired per participant, and repeated scan acquisitions were not performed. Consequently, test–retest repeatability and intra-session mandibular variability were not assessed and may represent an additional source of variation that was not captured within the present study design.

Comparison with traditional and alternative digital methods further highlights the method-specific nature of occlusal analysis outcomes. Research employing articulating paper has consistently shown that material properties tend to increase the number of recorded contact points, producing broader distributions than those observed in IOS-derived occlusal maps [23,24]. Conversely, digital force-based systems such as T-Scan provide highly sensitive temporal and force-distribution data, but are influenced by sensor thickness, calibration, and operator technique, and therefore differ fundamentally in data acquisition and output compared with static IOS-derived occlusal contact distance maps [11].

These distinctions underline that static occlusal contact analysis derived from IOSs should not be interpreted as a surrogate for dynamic occlusal force quantification, but rather as a method-specific visualization of spatial contact distribution obtained under controlled conditions.

The methodological simplicity employed in this study—using readily accessible commercial hardware and open-source image segmentation software—was intentional, aiming to replicate a realistic clinical workflow that does not rely on specialized equipment. The use of GIMP was specifically chosen to evaluate whether relative occlusal contact distance distributions could be quantified using universally available tools.

However, manual color-based segmentation is inherently operator dependent, and prior digital imaging research has demonstrated that open-source workflows may introduce measurable variability due to inconsistent thresholding and user-driven pixel selection [25]. This operator dependence represents an important limitation of open-source image analysis methods.

Accordingly, these findings underscore the potential value of automated, scanner-integrated analytic systems that apply standardized algorithms and reduce subjectivity. Studies investigating digital contact-area quantification similarly report that differences in thresholding, color-rendering algorithms, and region-of-interest selection can meaningfully affect calculated values [21,22]. Although the use of a standardized image field and a consistent segmentation protocol in the present study mitigated some of these sources of variability, further software integration would likely improve methodological robustness.

Several limitations of this study should be acknowledged when interpreting the findings. First, the sample size was relatively small (*n* = 15), which limits statistical power and may reduce the generalizability of the results. In addition, the study population consisted exclusively of healthy individuals with complete dentition and no signs or symptoms of temporomandibular disorders (TMDs) or other occlusal dysfunctions. While this homogeneity strengthened internal consistency by minimizing biological and functional variability, it also restricts the applicability of the findings to clinical scenarios involving compromised occlusion, symptomatic patients, or restorative and orthodontic cases, in which digital bite registration may behave differently.

Second, scans exhibiting interocclusal overlap greater than 0.1 mm were intentionally excluded to reduce alignment-related artifacts. Although this approach ensured a stable dataset under controlled conditions, it also means that the study did not evaluate the behavior of IOS-derived occlusal contact distance maps under less ideal circumstances. In routine clinical practice, bite scans may exceed this threshold due to patient movement, soft-tissue resilience, or operator-dependent scanning variability. Future investigations should therefore explore whether comparable distribution patterns can be obtained in more challenging, real-world clinical environments.

Third, although the image segmentation protocol was carefully standardized with fixed resolution, defined regions of interest, and consistent threshold parameters, the workflow remained manual and operator-dependent. Manual color-based segmentation, particularly when performed using open-source software such as GIMP, is inherently susceptible to variations in threshold selection, pixel interpretation, and user judgment. This limitation is consistent with prior reports indicating that non-proprietary digital imaging workflows may introduce measurable inter-operator and intra-operator variability. Greater automation within scanner-integrated analysis tools would likely improve consistency and reduce subjectivity.

An additional limitation is the absence of validation against a force-based occlusal analysis reference. IOS-derived occlusal contact distance maps reflect geometric proximity between virtual surfaces and do not provide information on occlusal force magnitude or timing. Although force-based systems such as T-Scan have been widely used to quantify occlusal load distribution, direct comparison with IOS-derived distance maps is constrained by fundamental differences in measurement principles, sensor characteristics, and output parameters [11]. As such, the present findings should be interpreted as reflecting consistency of distance-based visualization rather than biomechanical accuracy.

Another limitation relates to the use of a single scanner model and software environment. Intraoral scanners differ substantially in optical design, reconstruction algorithms, and occlusion-mapping heuristics, and results obtained with one system cannot be assumed to generalize across platforms. This heterogeneity is well documented in the literature and continues to limit meaningful cross-device comparison of digitally derived occlusal contact representations.

Finally, the cross-sectional design restricts conclusions regarding the temporal stability of digital bite registrations. The present study evaluated CR and MIP at a single time point, without repeated scans to assess intra-session or iner-session variability. Longitudinal investigations incorporating repeated registrations and correlation with clinical outcomes are needed to further elucidate the stability and clinical relevance of IOS-derived occlusal contact distance maps.

## 5. Conclusions

Under controlled scanning conditions with limited interocclusal overlap, intraoral = scanner-derived occlusal contact distance maps exhibited comparable distribution patterns between centric relation and maximal intercuspation. These findings indicate consistent distance-based occlusal visualization outcomes when identical acquisition and image-processing protocols are applied.

Within the methodological constraints of the present study, the results support the use of intraoral scanning for comparative assessment of occlusal contact distance distributions. Further investigations incorporating repeated registrations, different scanner systems, and force-based reference methods are warranted to better define the stability and clinical relevance of IOS-derived occlusal contact visualizations.

## Figures and Tables

**Table 1:** Inclusion and Exclusion Criteria for Study Participants.

Inclusion Criteria	Exclusion Criteria
Adults aged ≥ 18 years	Current or previous orthodontic treatment or fixed retention
Complete permanent dentition (excluding third molars)	Clinically significant malocclusions (e.g., Class II div. 2, Class III, crossbite, open bite)
No clinical signs or history of temporomandibular disorders (TMDs)	Missing posterior teeth affecting occlusal stability
Absence of occlusal dysfunction or orofacial pain	Removable or fixed prosthodontic appliances
No prosthodontic restorations or extensive dental work	History of jaw trauma or orthognathic surgery
Ability to understand and provide informed consent	Signs of bruxism or other parafunctional habits
Successful acquisition of CR and MIP scans with ≤0.1 mm occlusal overlap	Low-quality scans or occlusion maps with >0.1 mm interocclusal deviation

**Table 2:** Summary of occlusal contact distance category area percentages and paired *t*-test results.

Occlusal Contact Distance Category	CR Mean (%)	CR SD (%)	MIP Mean (%)	MIP SD (%)	Mean Diff (%)	95% CI Low (%)	95% CI High (%)	t-Value	*p*-Value	Cohen’s d
Very close	8.6	13.3	5.9	8.3	2.7	−3.6	8.9	0.8	0.4	0.2
Moderately close	44.8	9.8	44.7	8.7	0.0	−6.9	7.0	0.1	1.0	0.0
Distant	46.6	4.6	49.3	10.2	−2.7	−7.9	2.5	−1.0	0.3	−0.3

## Data Availability

The original contributions presented in this study are included in the article. Further inquiries can be directed to the corresponding author.
